# Supplementary material for: Causal Relationship between Aging and Anorexia Nervosa: A White-Matter-Microstructure-Mediated Mendelian Randomization Analysis
Source: Biomedicines. 2024 Aug 16;12(8):1874. doi: 10.3390/biomedicines12081874 (PMC11351342; doi:10.3390/biomedicines12081874)
Supplement: Supplementary file 1 [file biomedicines-12-01874-s001.zip › Supplementary Figure/Figure S8/Leaveoneout plot of MR analysis for TL to ubm-b-1931.pdf]

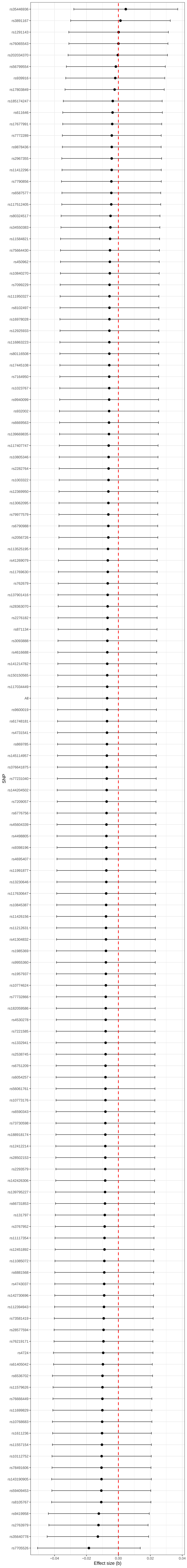

rs35446936
rs3891167
rs1291143
rs76065543
rs202034370
rs56799554
rs939916
rs17803849
rs185174247
rs611646
rs17677991
rs7772289
rs9878436
rs2967355
rs11412296
rs7790856
rs6587577
rs117512405
rs80324517
rs34550383
rs11584821
rs75664430
rs450962
rs10840270
rs7099229
rs111950327
rs8102497
rs16978028
rs12925933
rs116863223
rs80116508
rs17445106
rs7164950
rs1023767
rs9940099
rs932002
rs6669563
rs139669835
rs117407747
rs10805346
rs2282764
rs1003322
rs12369950
rs13062095
rs79977579
rs6790988
rs2056726
rs113525195
rs41269079
rs11769630
rs762679
rs137901416
rs28363070
rs2276182
rs871134
rs3093888
rs4616688
rs141214782
rs150150565
rs117034449
All
rs9600019
rs61748181
rs4731541
rs869785
rs145114957
rs376641875
rs77231040
rs144204502
rs7209057
rs6776756
rs45604339
rs4498805
rs9398196
rs4695407
rs11991877
rs13230646
rs117630647
rs10845387
rs11426156
rs11212631
rs41304832
rs1985369
rs9955360
rs1957937
rs10774624
rs77732866
rs182059586
rs4530278
rs7221585
rs1332941
rs2538745
rs6751209
rs6054257
rs56061761
rs10773176
rs6590343
rs73730598
rs188918174
rs12412214
rs28502153
rs2293579
rs142426306
rs139795227
rs66731853
rs131797
rs3767952
rs11117354
rs12451892
rs11085072
rs6881568
rs4743037
rs142730696
rs112394943
rs73581419
rs28577594
rs76219171
rs4724
rs61405042
rs6536702
rs11579626
rs76666449
rs11699829
rs10768683
rs1611236
rs11557154
rs10112752
rs78491606
rs143190905
rs59409453
rs8105767
rs9419958
rs2763979
rs35640778
rs7705526
SNP
-0.04
-0.02
0.00
0.02
0.04
Effect size (b)
